# Computed tomography enterography-based deep learning radiomics to predict stratified healing in patients with Crohn’s disease: a multicenter study

**DOI:** 10.1186/s13244-024-01854-x

**Published:** 2024-11-15

**Authors:** Chao Zhu, Kaicai Liu, Chang Rong, Chuanbin Wang, Xiaomin Zheng, Shuai Li, Shihui Wang, Jing Hu, Jianying Li, Xingwang Wu

**Affiliations:** 1 Department of Radiology, The First Affiliated Hospital of Wannan Medical College, Wuhu, 241000 People’s Republic of China; 2 Department of Radiology, The First Affiliated Hospital of Anhui Medical University, Hefei, 230022 People’s Republic of China; 3 Department of Interventional Radiology, The First Affiliated Hospital of USTC, Division of Life Sciences and Medicine, University of Science and Technology of China, Hefei, 230001 People’s Republic of China; 4 Department of Gastroenterology, The First Affiliated Hospital of Anhui Medical University, Hefei, 230022 People’s Republic of China; 5 CT Research Center, GE Healthcare China, Shanghai, 210000 People’s Republic of China

**Keywords:** Crohn’s disease, Mucosal healing, Transmural healing, Radiomics, Deep learning

## Abstract

**Objectives:**

This study developed a deep learning radiomics (DLR) model utilizing baseline computed tomography enterography (CTE) to non-invasively predict stratified healing in Crohn’s disease (CD) patients following infliximab (IFX) treatment.

**Methods:**

The study included 246 CD patients diagnosed at three hospitals. From the first two hospitals, 202 patients were randomly divided into a training cohort (*n* = 141) and a testing cohort (*n* = 61) in a 7:3 ratio. The remaining 44 patients from the third hospital served as the validation cohort. Radiomics and deep learning features were extracted from both the active lesion wall and mesenteric adipose tissue. The most valuable features were selected using univariate analysis and least absolute shrinkage and selection operator (LASSO) regression. Multivariate logistic regression was then employed to construct the radiomics, deep learning, and DLR models. Model performance was evaluated using receiver operating characteristic (ROC) curves.

**Results:**

The DLR model achieved an area under the ROC curve (AUC) of 0.948 (95% CI: 0.916–0.980), 0.889 (95% CI: 0.803–0.975), and 0.938 (95% CI: 0.868–1.000) in the training, testing, and validation cohorts, respectively in predicting mucosal healing (MH). Furthermore, the diagnostic performance of DLR model in predicting transmural healing (TH) was 0.856 (95% CI: 0.776–0.935).

**Conclusions:**

We have developed a DLR model based on the radiomics and deep learning features of baseline CTE to predict stratified healing (MH and TH) in CD patients following IFX treatment with high accuracies in both testing and external cohorts.

**Critical relevance statement:**

The deep learning radiomics model developed in our study, along with the nomogram, can intuitively, accurately, and non-invasively predict stratified healing at baseline CT enterography.

**Key Points:**

- Early prediction of mucosal and transmural healing in Crohn’s Disease patients is beneficial for treatment planning.
- This model demonstrated excellent performance in predicting mucosal healing and had a diagnostic performance in predicting transmural healing of 0.856.
- CT enterography images of active lesion walls and mesenteric adipose tissue exhibit an association with stratified healing in Crohn’s disease patients.

**Graphical Abstract:**

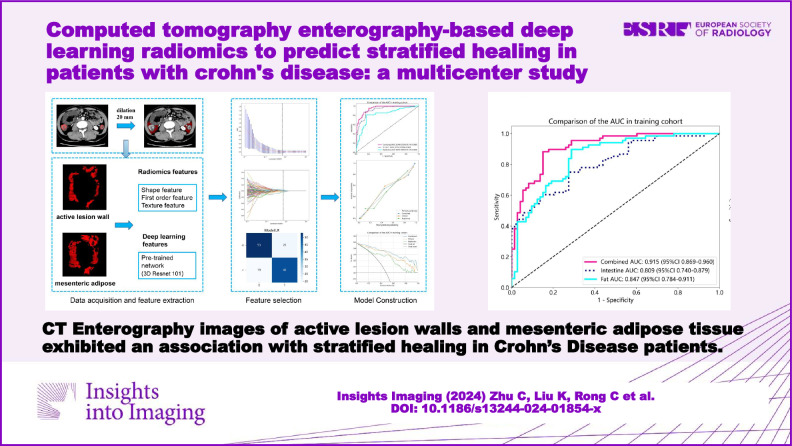

## Introduction

Crohn’s disease (CD) is a chronic, relapsing condition with a rising incidence worldwide, leading to various complications and imposing significant physical and psychological burdens on patients [1, 2]. Current treatment strategies aim for deep and sustained remission, to prevent complications, such as surgery, and halt disease progression. Central to these strategies is the early introduction of biologic therapy in high-risk patients, combined with stringent disease monitoring. Tumor necrosis factor (TNF) plays a crucial role in the pathogenesis of CD, and anti-TNF agents, such as infliximab (IFX), effectively induce clinical remission and promote mucosal healing (MH) and transmural healing (TH) in CD patients [3–5]. Early prediction of MH and TH achievement following IFX treatment in CD patients allows timely intervention and personalized treatment. Studies have shown that predictive biomarkers of MH and TH may continue to improve the prognosis of patients with CD [6, 7]. Therefore, developing biomarkers capable of predicting treatment response is becoming increasingly important for personalized therapeutic decision-making [8].

Computed tomography enterography (CTE) can reveal changes in both internal and external structures of the intestines, as well as associated complications [9]. While CTE effectively reflects the characteristics of CD, many internal details in the images are not discernible to the naked eye but can potentially be captured through radiomics [10]. Deep learning radiomics (DLR) is an emerging approach that uses supervised learning to extract quantitative and high-throughput features from medical images [11–13]. A recent study developed and validated a CT-based DLR model to predict early response to neoadjuvant chemotherapy in patients with locally advanced gastric cancer. This DLR model, which integrated imaging features and clinical factors, demonstrated good performance in predicting therapeutic efficacy and clinical outcomes, thus providing valuable information for personalized treatment [14]. While these DLR analyses have been applied in oncology, the application of DLR analysis in predicting therapeutic outcomes in CD remains to be further explored.

Our study aims to demonstrate the potential of a CTE-based DLR model for the early prediction of stratified healing (mucosal healing and transmural healing) following IFX treatment in CD patients. We developed a combined model through DLR analysis using CTE images of active lesion walls and mesenteric adipose tissue to non-invasively predict stratified healing in CD patients.

## Materials and methods

### Patients and study design

This retrospective study was approved by the Ethics Committees of all three participating hospitals, and the requirement for patient written informed consent was waived. The inclusion criteria were: (1) patients diagnosed with CD; (2) colonoscopy and CTE performed within 1 month prior to IFX treatment; (3) no prior biologic drug therapy; (4) CD lesions primarily located in the colon and terminal ileum; and (5) no abdominal and perianal abscesses. The exclusion criteria were: (1) not undergoing an endoscopic review 6–9 months after IFX treatment; (2) history of resection of the colon or terminal ileum; and (3) poor quality of CTE image interpretation.

**Fig. 1:**
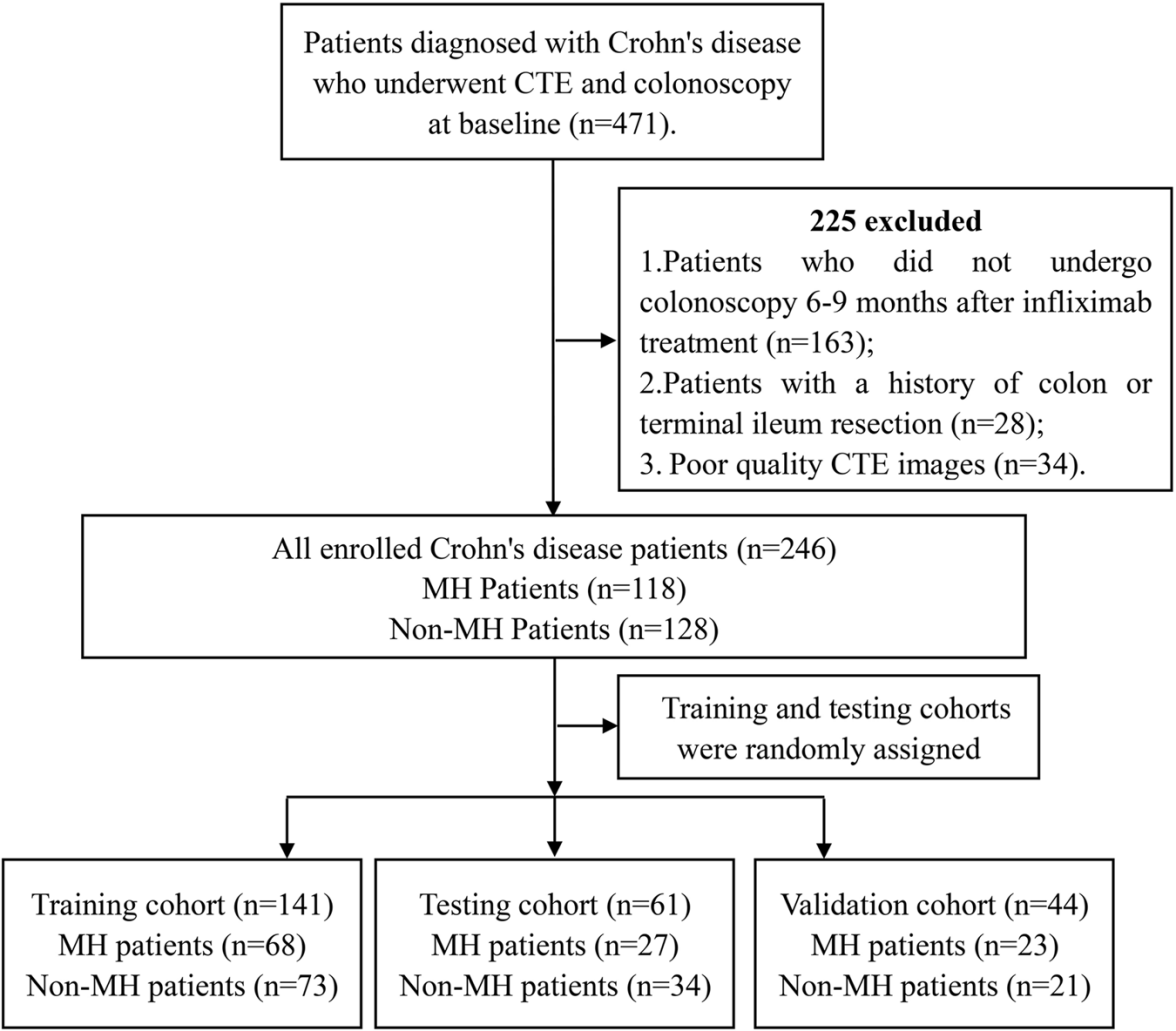
Flowchart of inclusion and exclusion criteria for the study population

We retrospectively reviewed the baseline clinical data and CTE images from 471 patients diagnosed with CD between March 2016 and December 2022 at three hospitals. A total of 246 patients with CD were included after exclusion by the above exclusion criteria. The 202 patients with CD from two hospitals were randomly divided into a training cohort (*n* = 141) and a testing cohort (*n* = 61) in a ratio of 7:3, and 44 patients with CD from the third hospital were used as a validation cohort (*n* = 44). The study flowchart is illustrated in Fig. 1.

The diagnosis of CD was based on a combination of clinical, endoscopic, stool, biochemical, and histological investigations [15]. CD patients were treated with IFX intravenously at a dose of 5 mg/kg at weeks 0, 2, and 6, followed by intravenous injections every 8 weeks. All enrolled patients underwent CTE and colonoscopy at baseline and had an endoscopic review 6–9 months after starting the IFX treatment [16]. Two gastroenterologists, each with over 15 years of experience in endoscopy, performed the endoscopic procedures and assessed the mucosal condition of each patient.

Simple Endoscopic Score for CD (SES-CD) is a widely recognized system for assessing MH in the colon and terminal ileum in CD patients. The terminal ileum and colon are divided into the following segments: terminal ileum, right colon, transverse colon, left colon, and rectum. Each segment’s inflammatory ulceration is assessed separately, and the scores are summed to obtain a total score. MH is defined as the disappearance of inflammatory ulcers and an SES-CD score of less than 3 in patients who had inflammatory ulcers in the terminal ileum and colon before treatment.

Our study first evaluated the predictive value of the baseline CTE-based DLR model for MH across the training, testing, and validation cohorts. Subsequently, within the MH group, patients were further classified into TH and non-TH groups to assess the DLR model’s predictive value for TH.

### CTE image acquisition

All enrolled patients underwent standardized bowel preparation before the computed tomography enterography (CTE) examination. Patients fasted for 12 h prior to the examination and consumed 500 mL of isotonic fluid at 45, 30, and 15 min before the scan. The three hospitals used either the GE Revolution CT or GE Optima CT680 for the CTE examinations. The scanning parameters were as follows: tube voltage of 120 kV, tube current of 150–300 mA, acquisition matrix of 512 × 512, pitch of 1.375:1, layer thickness and spacing of 5 mm. A nonionic contrast agent, iohexol (320 mg/mL), was injected peripherally at a dose of 1.5 mL/kg body weight and a flow rate of 3.0 mL/s.

### CTE image analysis, outlining and radiomics feature extraction

**Fig. 2:**
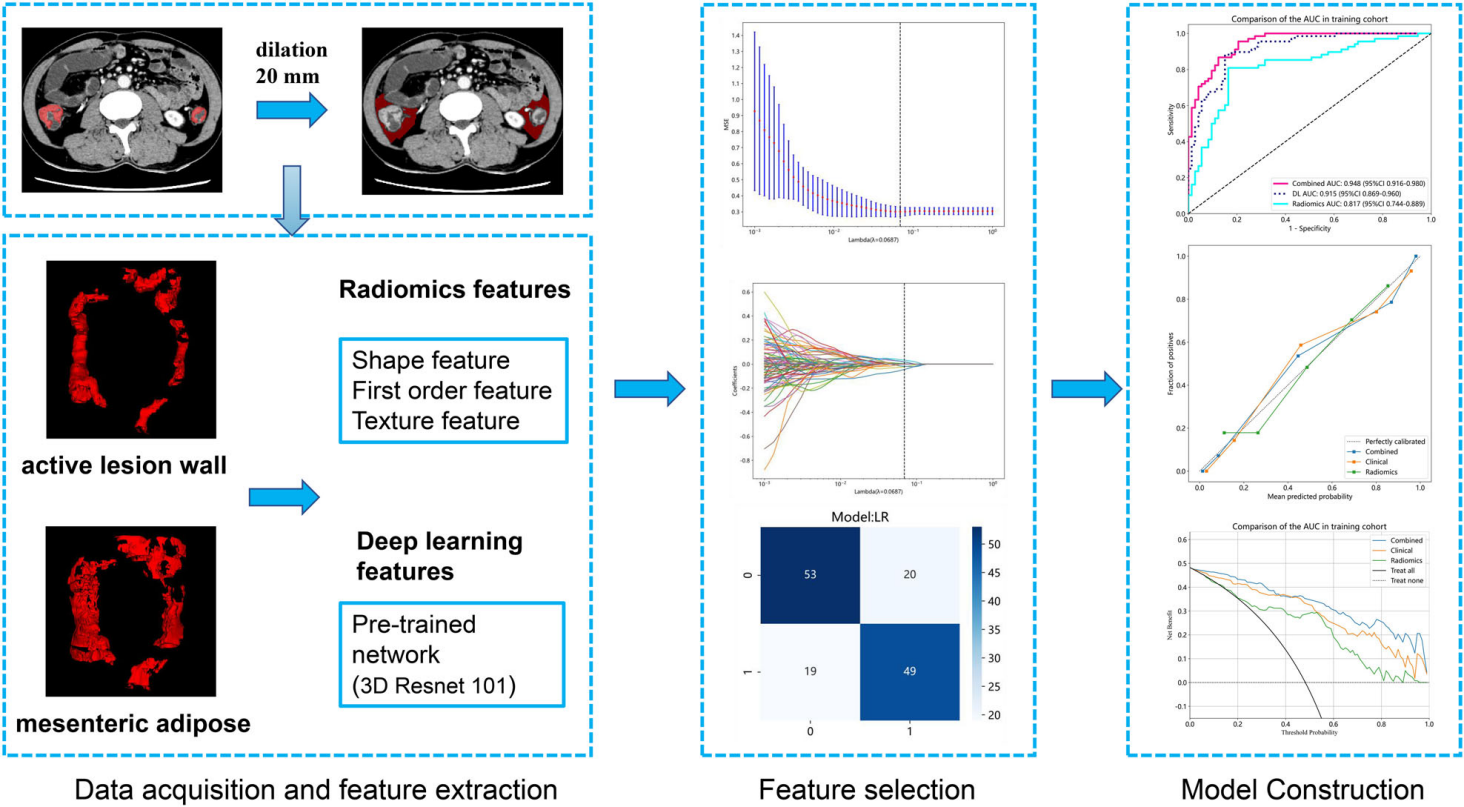
The radiomics flowchart of the study. Segmenting active lesions on the intestinal wall from CTE images in three dimensions, we employ a Python-based automatic dilation algorithm to expand the mesenteric fat tissue surrounding the active lesion-intestinal wall by 20 mm. We extract radiomic features and deep learning features separately from the intestinal wall and mesenteric fat. Feature selection and predictive model construction are carried out using machine learning methods. The efficacy of the model is evaluated using receiver operating characteristic (ROC) curves, calibration curves, and decision curve analysis (DCA)

To predict MH in the colon and terminal ileum of CD patients, two radiologists, each with over 5 years of experience in diagnostic abdominal imaging, independently reviewed the baseline CTE images. They were blinded to the post-treatment MH and TH outcomes. Volume of interest (VOI) was selected based on the following criteria: (1) The terminal ileum and colon lesions with inflammatory ulcers on the mucosal surfaces; (2) diseased bowel walls with thickening (> 3 mm) and hyperenhancement; (3) diseased bowel walls with inflammatory perforating complications (excluding abscesses) or active inflammation in the adjacent mesentery; and (4) bowel lumens where the VOI included the active lesion wall but excluded the lumen. The radiologists used ITK-SNAP 3.8.0 software for the three-dimensional (3D) outlining of these active diseased segments on baseline bowel phase CTE images (VOI1) (Fig. 2).

The inter- and intra-class correlation coefficients (ICCs) were used to evaluate the consistency of the outlining results between the two radiologists. CTE images of 30 patients were randomly selected for VOI outlining by both radiologists. Two weeks later, the same 30 patients’ CTE images were outlined again by radiologist 1. To ensure the reliability of the radiomics features, only features with ICCs greater than 0.75 were selected as candidate radiomics features. Radiologist 1 then performed VOI outlining for the remaining images. An automated expansion algorithm based on Python 3.6.8 software was subsequently used to obtain a VOI of the mesenteric adipose tissue (VOI2), expanding 20 mm around the center of VOI1 (Fig. 2). Mesenteric adipose tissue was defined as peristomal adipose tissue with a density of −45 to −195 Hounsfield units [17]. To avoid including adjacent intestinal lumens in the outward expansion, the range of VOI2 was manually adjusted.

To standardize the CTE images and mitigate the impact of different scanners and acquisition parameters on radiomics features, all images were resampled to a uniform voxel size of 1 × 1 × 1 mm³ before feature extraction. Using the PyRadiomics 3.0.1 package within Python 3.6.8 software, a total of 1834 radiomics features were extracted from VOI1 and VOI2 of each patient.

### Deep learning feature extraction

In our study, a 3D ResNet101 network architecture was utilized. The VOIs were selected from the original images and resized to 112 × 112 × 112 for input. The model training process involved updating the network weights using a cross-entropy loss function for the prediction task. Two 3D models were trained separately for the active diseased wall (VOI1) and mesenteric adipose tissue (VOI2). These two models were then used to extract DL features from each VOI. A total of 2048 deep learning (DL) features were extracted from the penultimate layer (avgpool layer) of the 3D model architecture. All data operations were performed using Python 3.6.8. The PyTorch framework was employed to train the model on an NVIDIA RTX 3070 Ti graphics processor units. Network optimization was conducted using the Adam optimizer with a learning rate of 0.001. The training process was carried out for 100 epochs with a batch size of 4.

### Inclusion of clinical characteristics

Table 1 outlines the clinical baseline characteristics of the enrolled CD patients. Clinical baseline data from the training cohort were first analyzed using univariate analysis. Clinical factors with *p* < 0.05 were then introduced into a multivariate logistic regression analysis. Clinical factors with *p* < 0.05 in the multivariate logistic regression analysis were identified as independent predictors.

**Table 1:** Clinical factors of MH and non-MH groups in the training, testing, and validation cohorts

Clinical features	Training cohort (*n* = 141)			Testing cohort (*n* = 61)			Validation cohort (*n* = 44)		
	MH	Non-MH	*p*-value	MH	Non-MH	*p*-value	MH	Non-MH	*p*-value
Age, years, (mean ± SD)	27.0 ± 9.0	28.6 ± 11.5	0.352	27.9 ± 10.6	29.9 ± 8.9	0.413	30.3 ± 11.6	22.6 ± 7.4	**0.013**
Gender, *n* (%)			0.076			0.071			0.235
Female	15 (22.1%)	26 (35.6%)		4 (14.8%)	12 (35.3%)		5 (21.7%)	8 (38.1%)	
Male	53 (77.9%)	47 (64.4%)		23 (85.2%)	22 (64.7%)		18 (78.3%)	13 (61.9%)	
Disease duration (months), median (IQR)	12 (4.8, 27)	12 (6, 26)	0.878	12 (6, 30)	12 (6, 36)	0.667	5 (2, 12)	12 (12, 60)	**0.001**
Montreal location, *n* (%)			1.000			0.787			1.000
Ileal	0 (0%)	0 (0%)		0 (0%)	0 (0%)		0 (0%)	0 (0%)	
Colonic	4 (5.9%)	4 (5.5%)		3 (11.1%)	2 (5.9%)		2 (8.7%)	2 (9.5%)	
Ileocolonic	64 (94.1%)	69 (94.5%)		24 (88.9%)	32 (94.1%)		21 (91.3%)	19 (90.5%)	
Penetrating, *n* (%)	4 (5.9%)	1 (1.4%)	0.321	4 (14.8%)	0 (0%)	0.072	0 (0%)	1 (4.8%)	0.477
Structuring, *n* (%)	4 (5.9%)	9 (12.3%)	0.186	1 (3.7%)	4 (11.8%)	0.503	1 (4.3%)	4 (19%)	0.290
Smoking status, *n* (%)	1 (1.5%)	4 (5.5%)	0.406	1 (3.7%)	1 (2.9%)	1.000	2 (8.7%)	0 (0%)	0.510
Serum CRP at baseline (mg/dL), median (IQR)	7.0 (3.1, 19.2)	14.4 (3.8, 35.6)	**0.030**	9.7 (3.3, 23.7)	12.5 (3.8, 28.3)	0.586	17.6 (6.4, 23.5)	17.6 (5.8, 40)	0.474
ESR at baseline (mm/h), median (IQR)	27 (13, 28.3)	34 (19, 39)	**0.021**	27 (21.5, 40)	32 (25, 34)	0.636	37 (28, 44)	34 (23, 42)	0.443
Serum albumin (g/dL) (mean ± SD)	39.1 ± 4.7	36.8 ± 5.0	**0.006**	39.1 ± 4.3	37.0 ± 5.2	0.091	37.4 ± 4.8	36.2 ± 6.2	0.489
WBC (× 10^9^ cells per L) (mean ± SD)	6.6 ± 2.0	6.6 ± 1.8	0.795	7.1 ± 2.6	6.5 ± 1.8	0.265	7.4 ± 2.1	7.2 ± 2.2	0.760
Previous steroids or immunomodulators use	21 (14.9%)	25 (17.7%)	0.670	9 (14.8%)	14 (23%)	0.530	4 (17.4%)	8 (38.1%)	0.124
HBI at baseline, median (IQR)	6 (5, 8)	7 (6, 8)	0.211	6 (5.5, 8)	7 (5.25, 9)	0.475	7 (5, 8)	8 (5, 10)	0.140
SES-CD at baseline, median (IQR)	15.5 (9, 26)	16 (12, 28)	0.379	22 (9, 28)	13 (8, 28)	0.423	14 (9, 20)	10 (8, 18)	0.239

Bold font indicates *p* < 0.05

*CRP* C-reactive protein *ESR* erythrocyte sedimentation rate, *WBC* white blood cell, *MH* mucosal healing, *HBI* Harvey-Bradshaw Index, *SES-CD* Simple Endoscopic Score for CD

### Construction of the radiomics and deep learning signatures

First, radiomics features with ICCs > 0.75 in the training cohort were retained. Next, radiomics and DL features with *p* < 0.05 were selected through univariate analysis. The most valuable radiomics and DL features were then identified using the least absolute shrinkage and selection operator (LASSO). Based on these selected features, radiomics and DL signatures were constructed. The diagnostic efficacies of these signatures in predicting MH were evaluated in the training, testing, and validation cohorts using AUC values under the ROC curve.

### DLR model construction

Radiomics and DL signatures were combined to build DLR model using multivariate logistic regression. The efficacies of the DLR model in predicting MH were evaluated in the training, testing, and validation cohorts using AUC values under the ROC curve. The net reclassification improvement (NRI) and integrated discrimination improvement (IDI) indices were used to quantify the enhancement of the radiomics signature with the inclusion of the deep learning signature. Calibration curves were employed to assess the agreement between the predicted probabilities of the DLR model and the actual outcomes. Decision curve analysis (DCA) evaluated the net clinical benefit of the DLR model in predicting MH across a reasonable range of threshold probabilities. The DeLong test was used to compare the diagnostic performance of the radiomics signature, the DL signature, and the DLR model.

### TH outcomes based on DLR model

Our study further evaluated the model’s ability to predict the TH outcomes. We collected data from CD patients who underwent both endoscopy and CTE or MRE examinations 6–9 months after IFX treatment. TH is defined as: based on MH, all ileocolonic segments have a wall thickness of less than 3 mm, with no ulcers, edema, enhancement, or complications [18, 19]. The diagnostic efficacy of DLR model in predicting TH was evaluated using AUC values under the ROC curve.

### Statistical analysis

Statistical analyses were conducted using SPSS 25.0, Python 3.6.8, and R 4.2.2 software. Measurements following a normal distribution were expressed as mean ± standard deviation, while those with a skewed distribution were presented as medians. Count data were expressed as the number of cases (percentage). For the comparison of measurement data, the independent samples *t*-test (for normally distributed data) or the Mann–Whitney *U* test (for skewed distribution) was used. The chi-square test or Fisher’s exact test was employed for count data comparisons. A *p*-value less than 0.05 was considered statistically significant.

## Results

### Patient characteristics

A total of 246 CD patients were enrolled in this study, with 118 achieving MH after IFX treatment. The clinical baseline data for the MH and non-MH groups in the training, testing, and validation cohorts are detailed in Table 1. In the training cohort, the univariate analysis of clinical characteristics showed that the serum CRP level was significantly lower in the MH group (7 mg/dL) than in the non-MH group (14.4 mg/dL) (*p* = 0.03). ESR was significantly lower in the MH group (27 mm/h) than in the non-MH group (34 mm/h) (*p* = 0.021). The serum albumin level was significantly lower in the MH group (39.1 g/dL) was significantly lower than that of the non-MH group (36.8 g/dL) (*p* = 0.006). However, the multivariate logistic regression analysis showed that none of these clinical characteristics was the independent predictor of MH (*p* ≥ 0.05).

### Radiomics signature establishment

Based on the ICCs, univariate, and LASSO regression analyses, we retained 13 radiomics features from the active diseased intestinal wall (VOI1) and 7 radiomics features from the mesenteric adipose tissue surrounding the diseased intestinal wall (VOI2) in the training cohort of CD patients. Using these radiomics features, we constructed a radiomics signature. The AUCs of radiomics signature in predicting MH were 0.817 (95% CI: 0.744–0.889) in the training cohort, 0.797 (95% CI: 0.683–0.911) in the testing cohort, and 0.822 (95% CI: 0.699–0.945) in the validation cohort. Table 2 displays the predictive efficacies of using radiomics signature across these three cohorts.

**Table 2:** Performance of the radiomics signature, DL signature, and DLR model in the training, testing, and validation cohorts

Models	Training cohort (*n* = 141)				Testing cohort (*n* = 61)				Validation cohort (*n* = 44)			
	AUC	SEN	SPE	ACC	AUC	SEN	SPE	ACC	AUC	SEN	SPE	ACC
Radiomics signature	0.817	0.836	0.809	0.823	0.797	0.676	0.815	0.738	0.822	0.810	0.739	0.773
DL signature	0.915	0.849	0.882	0.865	0.871	0.706	0.926	0.803	0.878	0.905	0.739	0.818
DLR	0.948	0.795	0.956	0.872	0.889	0.794	0.926	0.852	0.938	0.905	0.913	0.909

*AUC* area under the curve, *SEN* sensitivity, *SPE* specificity, *ACC* accuracy, *95% CI* 95% confidence interval, *DL* deep learning, *DLR* deep learning radiomics

### Deep learning signature establishment

Similar to the method used for selecting radiomics features above, we retained 19 DL features from VOI1 and 20 DL features from VOI2 in the training cohort of CD patients. Using these DL features, we constructed a DL signature. The AUCs of DL signature in predicting MH were 0.915 (95% CI: 0.869–0.960) in the training cohort, 0.871 (95% CI: 0.784–0.959) in the testing cohort, and 0.878 (95% CI: 0.776–0.980) in the validation cohort. Table 2 displays the predictive efficacies of using DL signature across these three cohorts.

### Deep learning radiomics model and nomogram establishment

Considering the predictive value of the DL signature, we determined its value-added to the radiomics signature in predicting MH through IDI and NRI. When combining the radiomics signature with the DL signature, we observed a large improvement in the predictive efficacy compared to using the radiomics signature alone. This improvement was statistically significant in IDI (training cohort: IDI = 0.324, 95% CI: 0.245–0.403, *p* < 0.05; testing cohort: IDI = 0.216, 95% CI: 0.112–0.320, *p* < 0.05; validation cohort: IDI = 0.335, 95% CI: 0.191–0.480, *p* < 0.05). This improvement was also statistically significant in NRI (training cohort: NRI = 0.555, 95% CI: 0.348–0.763, *p* < 0.05; testing cohort: NRI = 0.516, 95% CI: 0.211–0.821, *p* < 0.05; validation cohort: NRI = 0.495, 95% CI: 0.159–0.831, *p* < 0.05).

**Fig. 3:**
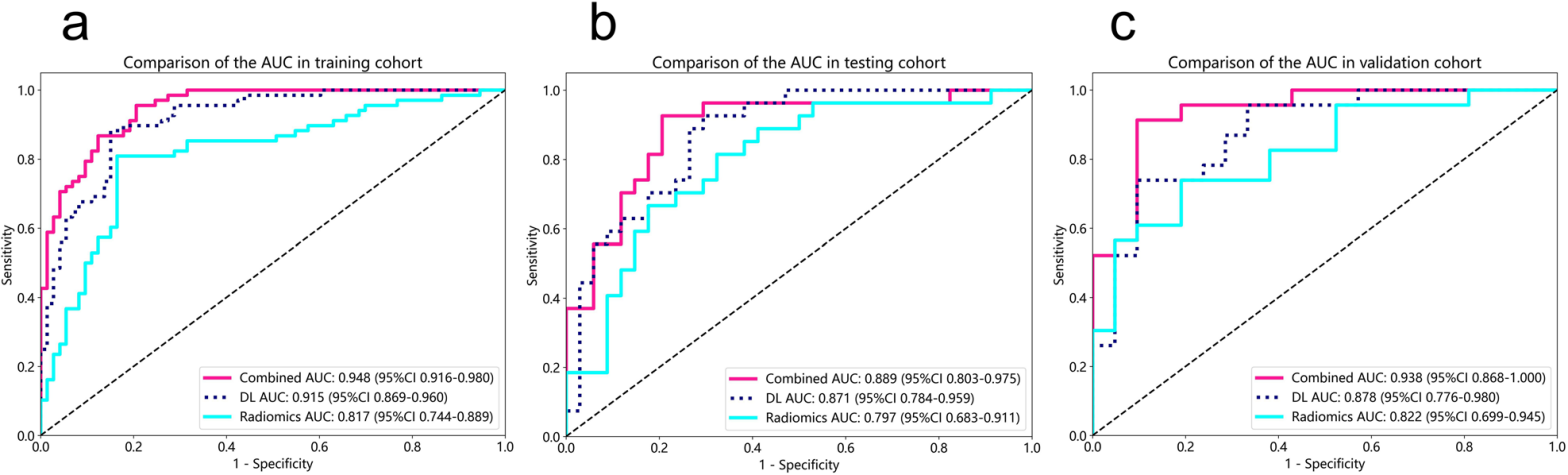
The receiver operating characteristic (ROC) curves for the radiomics model, deep learning model, and DLR (deep learning radiomics) model are evaluated in the training, testing, and validation cohorts. **a** ROC curves for the three models in the training cohort; **b** ROC curves for the three models in the testing cohort; **c** ROC curves for the three models in the validation cohort

**Fig. 4:**
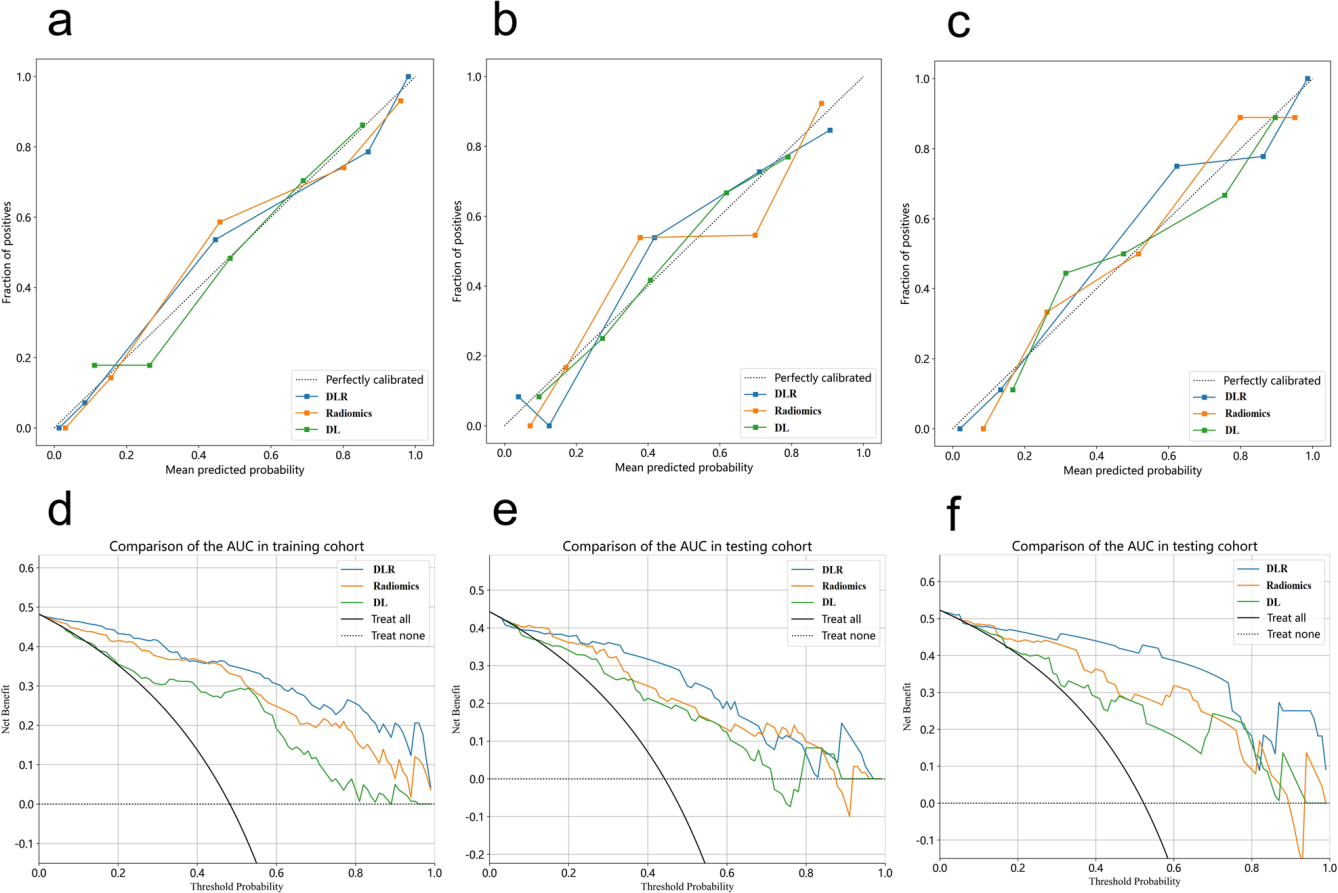
The radiomics model, deep learning model, and DLR (deep learning radiomics) model are subjected to calibration curve and decision curve analysis (DCA) in the training, testing, and validation cohorts. **a**, **d** Calibration curves and DCA curves for the three models in the training cohort. **b**, **e** Calibration curves and DCA curves for the three models in the testing cohort. **c**, **f** Calibration curves and DCA curves for the three models in the validation cohort

**Fig. 5:**
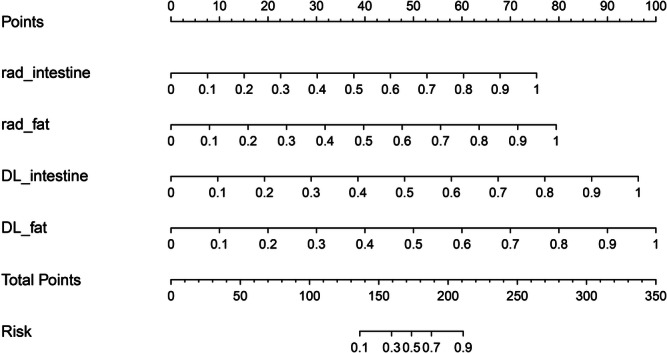
DLR (deep learning radiomics) nomogram. The predicted values of the radiomics model and deep learning model for active diseased intestinal wall, as well as the radiomics model and deep learning model for mesenteric fat tissue, can be transformed into quantitative scores along the integral axis. By summing up individual quantitative scores, the final total score is displayed on the total integral axis, and the corresponding predicted probability for mucosal healing is derived

The AUCs of the DLR model were 0.948 (95% CI: 0.916–0.980) in the training cohort, 0.889 (95% CI: 0.803–0.975) in the testing cohort, and 0.938 (95% CI: 0.868–1.000) in the validation cohort. Table 2 presents the diagnostic performance of the DLR model across the training, testing, and validation cohorts. Figure 3 displays the ROC curves for the three models, illustrating the excellent performance of the DLR model. Figure 4 shows the calibration and DCA curves for the three models. The calibration curves demonstrated that the predicted probabilities of the DLR model aligned well with the actual results. The DCA curves indicated that the DLR model provided a higher net clinical benefit in predicting MH compared to the radiomics and DL signatures across most reasonable threshold probability ranges. The DLR nomogram is shown in Fig. 5.

### Evaluation of the efficacy of deep learning radiomics model

The DeLong test was used to compare the DLR model, the radiomics signature, and the DL signature (Table 3). In the training cohort, the DLR model significantly outperformed both the radiomics signature and the DL signature (*p* = 0.001, 0.025, respectively). The DL signature also showed superior performance over the radiomics signature (*p* = 0.030). In the testing cohort, the DLR model demonstrated a significant improvement over the radiomics signature (*p* = 0.036). Similarly, in the validation cohort, the DLR model was significantly better than the radiomics signature (*p* = 0.025).

**Table 3:** DeLong test of the three models in the training, testing and validation cohorts

	Radiomics signature vs DL signature	Radiomics signature vs DLR model	DL signature vs DLR model
Training cohort	**0.030**	**0.001**	**0.025**
Testing cohort	0.270	**0.036**	0.587
Validation cohort	0.515	**0.025**	0.161

Bold font indicates *p* < 0.05

*DL* deep learning, *DLR* deep learning radiomics

### Predictive performance for TH patients

We further evaluated the ability of the DLR model to predict TH in CD patients after IFX treatment. Among the 118 patients who achieved MH, 86 CD patients underwent CTE or MRE examinations 6–9 months after IFX treatment. Of these, 21 (24.4%) patients achieved TH, 65 (75.6%) patients did not achieved TH. The DLR model demonstrated a diagnostic performance of 0.856 (95% CI: 0.776–0.935) in predicting TH.

## Discussion

Currently, IFX is crucial for achieving sustained clinical remission in CD patients. Our study developed a CTE-based DLR model to predict MH following IFX treatment. The DLR model was independently validated across a multicenter cohort, showing AUC values of 0.948, 0.889, and 0.938 in the training, testing, and validation cohorts, respectively. The DLR nomogram and DCA curve further confirmed its clinical applicability. Furthermore, The DLR model demonstrated excellent performance in predicting TH.

Clinical goals for treating CD include improving laboratory markers, achieving clinical symptom remission, and attaining MH. However, patients in clinical remission may still experience a loss of response and recurrence of symptoms [20, 21], with loss of response reported in approximately 23–46% of patients [22]. Therefore, the therapeutic goal for CD has shifted to MH and TH [23–25]. Currently, determining MH after IFX treatment relies on assessing mucosal ulcers, but endoscopy requires bowel preparation, sedation or anesthesia and carries risks such as intestinal perforation [26]. As a result, there is growing interest in finding non-invasive biomarkers to predict MH and TH. Predicting the therapeutic response to medications is crucial for selecting the most appropriate treatment, thereby improving clinical care and cost-effectiveness. This focus on individualized treatment aligns with the principles of precision medicine [27].

There are fewer studies predicting MH at baseline before IFX treatment. Bertani et al included 45 patients with CD treated with IFX, all of whom underwent colonoscopy at week 54 to assess MH. Serum oncostatin M and fecal calprotectin were collected before and after 14 weeks of treatment, respectively. Compared with fecal calprotectin, baseline serum oncostatin M is a promising biomarker for predicting MH and guiding treatment selection for CD [23]. It has been shown that 12-week clinical outcome and CRP, shorter disease duration, and non-smoking status are independent predictors of 12-month clinical outcome, while 12-week CRP, 24-week clinical remission, inflammatory parameters, and non-smoking are associated with MH [28]. In our univariate analysis of clinical characteristics, serum CRP, albumin, and ESR levels were significantly lower in the MH group than in the non-MH group. However, multivariate logistic regression analysis of these clinical characteristics showed that none was an independent predictor of MH. Therefore, we did not include clinical features in our final prediction model.

DLR technology is a high-throughput quantitative feature extraction technique that extracts and quantifies subtle imaging features related to potential disease heterogeneity beyond what the radiologist can detect visually. These features are then used in machine learning algorithms capable of predicting relevant outcomes. Previous studies have demonstrated the potential value of radiomics features of CTE images of the bowel wall of active lesions in predicting outcomes after IFX treatment in patients with CD [12, 29]. However, these studies often lack external validation, do not include mesenteric adipose tissue, and do not incorporate DL features. Our study demonstrated that both the radiomics model and the DL model based on active diseased intestinal wall and mesenteric adipose tissue achieved excellent predictive efficacy, including in the validation cohort. Our findings capability underscores the model’s potential to provide comprehensive insights into both mucosal and transmural healing, offering a more holistic approach to monitoring and managing CD treatment outcomes.

Our study has several limitations. First, to comprehensively predict MH in the colonic and terminal ileal segments of CD patients, we manually delineated the VOIs for all active lesions in these segments. Manual segmentation is time-consuming and labor-intensive. Although DL algorithms hold promise for automatic lesion segmentation, the complexity of intestinal segmentation poses challenges. Current studies report a Dice similarity coefficient of 0.824 for DL segmentation models in segmenting CD lesions [30], indicating a gap between the accuracy of automated and manual segmentation. Second, due to the low incidence of CD, our study sample size was relatively small, particularly for patients with TH. Future research should aim to increase the sample size to validate our findings.

## Conclusions

We developed a CTE-based DLR model combining both radiomics and deep learning features from both the active diseased bowel wall and mesenteric adipose tissue to predict MH following IFX treatment in patients with CD and achieved high accuracy. The predictive power of this DLR model was independently validated in an external cohort, demonstrating its intuitive, accurate, and non-invasive capability to predict MH and TH.

## Data Availability

The data underlying this article cannot be shared publicly due to the privacy of individuals who participated in the study. The data will be shared upon reasonable request to the corresponding author.

## Abbreviations

3D: Three-dimensional
AUC: Area under the ROC curve
CD: Crohn’s disease
CRP: C-reactive protein
CTE: Computed tomography enterography
DCA: Decision curve analysis
ICCs: Inter- and intra-class correlation coefficients
IDI: Integrated discrimination improvement
IFX: infliximab
LASSO: Least absolute shrinkage and selection operator
MH: Mucosal healing
NRI: Net reclassification improvement
ROC: Receiver operator characteristic
SES-CD: Simple Endoscopic Score for CD
TH: Transmural healing
TNF: Tumor necrosis factor
VOI: Volume of interest
